# Molecular Determination of Sex from Down and Feather in Wild and Reared Monomorphic and Dimorphic Birds at Juvenile Age

**DOI:** 10.3390/ani15060892

**Published:** 2025-03-20

**Authors:** Antonio Ciro Guaricci, Mario Cinone, Salvatore Desantis, Giovanni Michele Lacalandra, Maria Albrizio

**Affiliations:** 1 Department of Regenerative and Precision Medicine and Ionian Area (DiMePRe-J), University of Bari Aldo Moro, 70121 Bari, Italy; mario.cinone@uniba.it (M.C.); salvatore.desantis@uniba.it (S.D.); maria.albrizio@uniba.it (M.A.); 2 Department of Veterinary Medicine (DiMeV), University of Bari Aldo Moro, 70010 Valenzano, Italy; giovannimichele.lacalandra@uniba.it

**Keywords:** molecular sexing, monomorphic bird, dimorphic bird, chicks, RFLP-PCR, down, feather

## Abstract

The inability to distinguish males from females in young birds is a major obstacle for pair formation in reintroduction–restocking programs and commercial–amateur breeding. Several techniques are employed to address this problem, but not all of them are suitable for juvenile subjects. Among the various tests applied for sex determination, polymerase chain reaction (PCR) is one of the genetic tools that seems to be most effective (rapid, not invasive and cheaper). In this study, DNA was extracted from down and feathers to make the procedure less stressful for nestlings. The DNA was amplified by PCR, and the amplicon was subjected to the restriction endonucleases procedure when the gender was not clearly identified by PCR alone. One hundred and fifty-three avian species were sexed using this procedure, including 27 for the first time. In all the nestlings and juveniles tested, sex was correctly identified; in fact, all pairs that reached sexual maturity during this study gave offspring.

## Simple Summary

Interest in the breeding of feathered species has grown increasingly in recent years to the extent that early sexing has become necessary to ensure the formation of the pairs that will guarantee the birth of new birds. When sex identification must be carried out in very young individuals, sex is hardly recognizable not only in monomorphic species but also in dimorphic ones. The employment of molecular techniques is particularly necessary when the assessment of biometric parameters does not allow a clear sex identification. Recovery of DNA is mandatory when molecular techniques are used. In this paper, we demonstrate that down and feathers are suitable sources for DNA recovery without causing severe stress to the bird. This is a crucial aspect when dealing with nestlings. Using this procedure, we identified the gender of 153 avian species, including 27 for the first time.

## 1. Introduction

In recent years, bird breeding has found renewed interest in several countries, both for basic scientific reasons and because of the gradual spread of ornamental–amateur value species breeding. In addition, the number of projects for the reintroduction of threatened species [1] and programs for integrated conservation through the release of wild or captive-bred individuals [2] has grown enormously. About 60% of bird species are monomorphic [3], making it difficult to identify the sex of juvenile and adult individuals.

Among the various problems to be addressed in the context of breeding plans for native and/or exotic birds, the knowledge of sex has primary importance for the correct pairing of the subjects that will constitute the pair. The minimal or no sexual dimorphism in many avian species makes sex determination based on morphological features unreliable and virtually impossible in juvenile subjects [4]. This difficulty is also evident in newly hatched birds [5].

Sex can also be determined through acoustic venting [6], laparoscopy [7], steroid levels [8], karyotyping [9], differences in morphometric traits [10], or by comparing blood plasma protein profiles between males and females [11]. However, all these approaches are not always suitable due to the long time needed to perform some of them or because of invasiveness and high cost or inapplicability to monomorphic species.

By contrast, molecular methods for sex identification are more feasible. Starting from 1996, the heterogameticity of the females (they have a Z and a W chromosome) and the homogameticity of the males (they have two equal Z chromosomes) were employed to highlight the differences in the nucleotide sequences of specific genes. Some genes were proposed as markers of sex identification based on variations in their intron lengths. One of the most used was the Chromodomain-Helicase DNA Binding Protein 1 (CHD1) because of its sex-linked intron length showing slight differences between the Z and W chromosomes. Using a specific primer pair and PCR, an amplification product is obtained that migrates as a single band in ZZ males and as two bands in ZW females when electrophoresed in agarose gel under the effect of an electric field [12,13].

In recent years, many molecular methods have been developed and employed for sexing avian species: single-strand conformation polymorphism (SSCP), restriction fragment length polymorphism (RFLP), random amplified polymorphic DNA (RAPD), amplified fragment length polymorphism (AFLP), microsatellites, allele-specific PCR (AS-PCR), capillary electrophoresis, real-time quantitative PCR (qPCR), real-time PCR combined with melting curve analysis, high-resolution melting (HRM) analysis ([14] for review), and single nucleotide polymorphism (SNP) [15]. These techniques overcome the main animal health risk deriving from surgery. Molecular sexing is virtually applicable to any bird, regardless of age, and becomes the best choice if it is aimed at sexing born offspring. The PCR technique can also be combined with the amplicon digestion by restriction endonucleases (PCR-RFLP) when the size of the amplified product shows the same size in the two sexes [16,17,18]. Different sources such as blood, feathers, feces, eggshell membrane [14], and cells from buccal swabs can be used to obtain DNA [19]. In this study, the RFLP-associated PCR method was used when the amplicons of the *CHD*1 gene showed non-appreciable differences between the two sexes. The aim of this work was to demonstrate that primers P2 and P8, first used by Griffiths et al. [20], are suitable for sexing more than 150 avian species belonging to different orders when combined with RFLP in wild and reared chicks. The merit of this work was to provide those interested in bird sexing with the correct relationship between species and testing methods. Although feather picking may induce temporary discomfort or stress, the birds are ultimately released unharmed, so we have shown that breast feathers are an adequate source for extracting enough DNA, especially for juvenile subjects.

## 2. Materials and Methods

This research was conducted over a seven-year period. Adult individuals were initially tested to see whether the couple of primers employed in the PCRs were effective in amplifying the CHD1 gene in all bird species analyzed and in distinguishing between males and females. When the PCR product was obtained without differences between the two sexes, the restriction enzyme HaeIII was tested, and when the restriction fragments produced were also not able to distinguish males from females, the restriction enzyme Asp 700I was used. Subsequently, once the correct PCR approach was established for each species, young birds were sexed, and approximately 70% of the pairs formed produced offspring during this study. In total, about 3500 samples from 153 different bird species were analyzed.

### 2.1. Sample Collection

**Figure 1:**
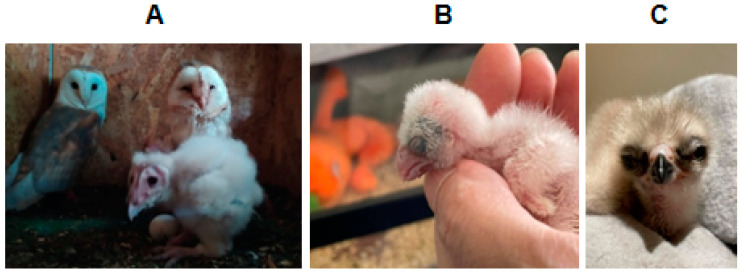
Representative images of adults and newborn birds sexed in this study: (**A**) *Tyto alba*; (**B**) *Falco naumanni*; and (**C**) *Buteo buteo*.

Three to four down or feathers retrieved from the chest of adults, newly hatched chicks or juvenile birds (Figure 1) were provided by breeders, ornithologists, wild avifauna rehabilitation centers, and zoos. Samples retrieved from the Mediterranean area were brought directly to our laboratory or arrived by mail and courier. In some cases, samples arrived even 2–4 weeks after collection. Upon arrival, down/feathers were analyzed immediately. All the suppliers were recommended to collect feathers and down directly from the birds, avoiding the recovery of those fallen on the ground or in the cages.

### 2.2. DNA Extraction

Pieces of calamus 1–2 mm long from three feathers or down were used as genomic DNA sources. Extraction was performed using a commercial kit (GenElute mammalian genomic DNA miniprep kit, Sigma-Aldrich, Milan, Italy) according to the manufacturer’s instructions with slight optimized modifications, such as the digestion of samples in lysis buffer T added with proteinase K 10 mg/mL at 56 °C overnight and the final elution of the DNA from the binding silica using only 100 μL of TE buffer. The obtained DNA was quantified by a UV spectrophotometer (Beckman Instruments Inc. Fullerton, CA, USA), its purity assessed (ratio A260/A280 = 1.8–2.0) and finally stored at −20 °C until PCR analysis.

### 2.3. PCR and RFLP Analysis

The polymerase chain reaction was performed using the P2-forward and P8-reverse primers (Sigma-Aldrich, Milan, Italy) proposed by Griffiths et al. [20] in 1998, in a volume of 50 μL with 100 ng of DNA, 1U Hot Master Taq polymerase (Eppendorf, Milan, Italy), 200 μM each dNTP and 1 X Taq-buffer. The amplification profile was the following: an initial denaturation step at 95 °C for 2 min, 35 cycles at 95 °C for 45 s, 48 °C for 45 s and 72 °C for 45 s, and a final elongation step at 72 °C for 5 min.

PCR products were run on 3% agarose gel in TAE buffer with 0.5 μg/mL ethidium bromide and a molecular weight marker (Eppendorf, Milan, Italy) to verify the dimensions of the resulting fragments. The gels were visualized under UV light with GelDoc 2000 apparatus (BioRad, Milan, Italy). The acquired images were analyzed to evaluate the sizes of the amplified products.

Restriction fragment length polymorphism analysis was conducted by digesting 15 μL of the PCR product with 5U of HaeIII enzyme (Roche Diagnostics, Milan, Italy) [16], 1 X corresponding digestion buffer in a total volume of 20 μL at 37 °C for 3 h; the samples were run on a 2% agarose gel and visualized under UV light with GelDoc 2000, as previously described. When the Hae III restriction enzyme was not useful for producing different-size fragments in the males and females, a new RFLP analysis was performed on another 15 μL aliquot of the same amplified product with 5 U of Asp 700I (Roche Diagnostics, Milan, Italy) [16] at the same conditions of incubation, electrophoresis, separation, and UV visualization.

## 3. Results

**Figure 2:**
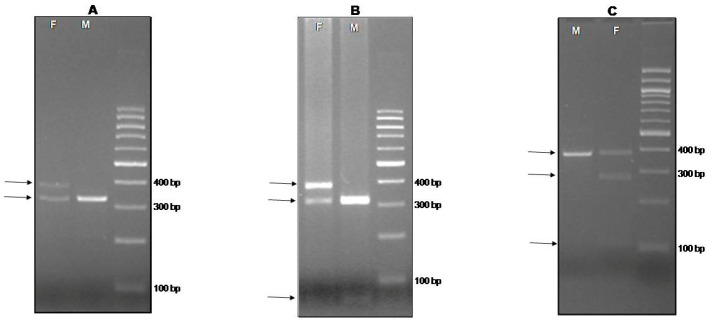
Representative molecular sexing of adult and juvenile birds. Panel (**A**), DNA from down or feather was amplified by PCR employing P2/P8 primers as in Griffiths et al. [20] and electrophoresed on 3% agarose gel. Panel (**B**), PCR product digested by HaeIII restriction enzyme. Panel (**C**), PCR product digested by Asp 700I restriction enzyme. In each panel, the number and size of each band allow the identification of the gender: females (F) are identified by two bands in panel (**A**) and three bands in panel (**B**) and (**C**); males (M) are identified by one band in panel (**A**) and (**C**) and two bands in panel (**B**). The arrow indicates the obtained amplicons, dimensions in base pair of the molecular weight marker bands are on right-hand side.

To test that the P2/P8 primer pair was capable of amplifying and discriminating juvenile males and females in all avian species belonging to the orders listed in Table 1, a preliminary analysis was conducted on adult individuals of known gender of the same species. For all samples received, even those collected up to one month before analysis, the extracted DNA was of good quality and in a concentration more than sufficient for subsequent analysis. In males, regardless of order and species, the amplicon consisted of a single band that was characterized by gel electrophoresis and whose approximate size is shown in Table 1 and Figure 2.

As for the females, the species belonging to the orders of Otidiformes, Podicipediformes, and Sphenisciformes showed a double amplicon of different sizes (shown in Table 1), so sex identification was easily performed by a simple PCR reaction followed by gel electrophoresis (Figure 2A).

All the other orders listed in Table 1 required the use of the PCR-RFLP approach for most species. All species were sexed by this technique, and the approximative size of the fragments resulting from the enzymatic digestion by HaeIII is listed in Table 1 and Figure 2, panel B.

In detail, in the order of Accipitriformes, the following species required PCR: *Cathartes aura*, *Gypohierax angolensis*, *Neophron percnopterus*, *Pernis apivorus*, and *Sarcoramphus papa*. All the others were sexed by HaeIII digestion. In the order of Anseriformes, HaeIII digestion was necessary for the species *Anas platyrhynchos.*

The same procedure was followed for the orders of Apodiformes (species *Apus apus*), Bucerotiformes (species *Aceros nipalensis*, and *Bucorvus leadbeateri*), Charadriiformes (species *Ichthyaetus melanocephalus*, *Himantopus Himantopus*, and *Vanellus vanellus*), Ciconiiformes (species *Ciconia ciconia*), Columbiformes (species *Streptopelia decaocto*), Coraciiformes (species *Merops apiaster*), Cuculiformes (species *Cuculus canorus*), for the order of Falconiformes (species *Falco vespertinus*, *Falco ardosiaceus*, *Falco biarmicus*, *Falco jugger*, *Falco naumanni*, and *Falco subbuteo*), Galliformes (species *Coturnix coturnix* and *Polypectron napoleonis*), Gruiformes (species *Balearica regulorum*, *Fulica atra*, and *Grus grus*), Passeriformes (species *Monticula solitarius*, *Oriolus oriolus*, *Parus major*, and *Serinus serinus*), Pelecaniformes (all species required PCR-RFLP except for *Egretta garzetta*), and Suliformes (the species *Phalacrocorax carbo*).

In the order of Strigiformes, the species *Strix aluco*, *Strix rufipes*, and *Strix uralensis* were sexed by simple PCR, while all the others were sexed by HaeIII digestion.

When even PCR-RFLP by HaeIII did not allow for the differentiation between females and males, the restriction enzyme Asp 700I was employed in the PCR-RFLP procedure (Figure 2, panel C). This approach was used to sex *Chauna chavaria* of the order Anseriformes and *Phoenicopterus chilensis* and *Phoenicopterus roseus* of the Phenicopteriformes order. The approximative sizes of the fragments produced after the enzymatic digestion of the amplicon are listed in Table 1.

**Figure 3:**
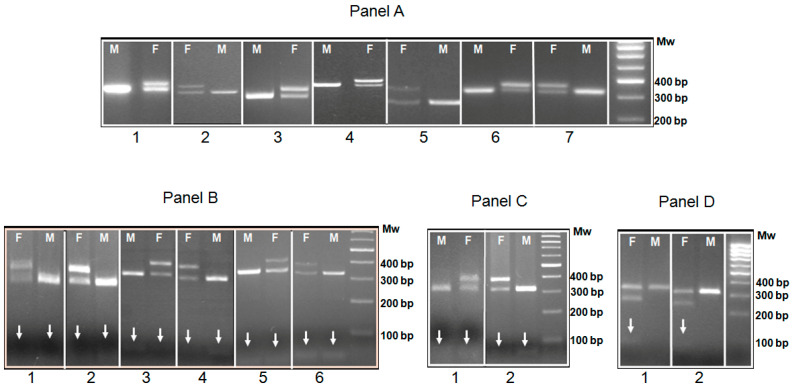
Representative molecular sexing of adult and juvenile birds belonging to species sexed for the first time in this study. In panel (**A**), species sexed only by PCR: 1 = *Trichoglossus moluccanus*, *Coleus monedula*; 2 = *Aythya fuligula*, *Strix rufipes*; 3 = *Chloris chloris*; 4 = *Gypohierax angolensis*; 5 = *Spinus magellanicus*; 6 = *Alectoris barbara*, *Spatula querquedula*; 7 = *Agapornis taranta*, *Pinicola enucleator*, *Netta rufina*, *Turdus iliacus*, *Turdus philomelos*, *Turdus viscivorus*, *Burhinus oedicnemus*. In panel (**B**), species sexed by HaeIII RFLP: 1 = *Oriolus oriolus*; 2 = *Serinus serinus*; 3 = *Pelecanus crispus*; 4 = *Ichthyaetus melanocephalus*, *Polypectron napoleonis*; 5 = *Falco ardosiaceus*; 6 = *Aceros nipalensis*. In panel (**C**), more species sexed by HaeIII RFLP: 1 = *Ixobrychus minutus*; 2 = *Monticola solitarius*. In panel (**D**), species sexed by Asp 700I RFLP: 1 = *Phoenicopterus chilensis*; 2 = *Chauna chavaria*. M = male, F = female, Mw = molecular weight marker, and the arrows indicate faint smaller bands. For approximative dimensions in the base pair of each band, refer to Table 1.

After defining the size of the amplicons and/or restriction fragments in each species, we subjected the DNA obtained from the down of young individuals to amplification. Every young subject was easily sexed, obtaining the expected amplicon or restriction fragment. By this approach, to the best of our knowledge, 27 new species (shaded row in Table 1 and Figure 3) were sexed for the first time.

## 4. Discussion

Phenotypic traits cannot help in the sex determination of monomorphic species. To overcome this problem, a molecular PCR-based test to define the gender of 153 different avian species was employed.

Sex identification in birds is of considerable importance for many reasons, ranging from understanding changes in sex ratio between generations or in specific geographic regions to assessing the health, behavior, and ecological dynamics of populations and facilitating reproduction in captivity. In birds, predicting sex is a difficult task because temperature or other environmental factors have no effect on avian sex determination or gonadal development as they influence these processes in non-avian reptiles, amphibians, and some fish [21].

Furthermore, early sex determination is mandatory for managing captive birds and improving breeding programs because it allows for precocious pair formation.

Considering this primary objective, this study evaluated the efficacy of DNA extraction from a small number of down collected easily and with slight pain from the chest of nestling or juvenile birds. Previous studies have shown that up to 10 ng/mL of DNA is obtained from adult small feathers. This DNA is also of good quality and virtually contamination-free because, during growth, feathers supplied with blood keep the DNA inside the stem, protecting it from potential degradation and microbial contamination [22]. In our case, using growing feathers from young individuals, the DNA concentration obtained was around 15 ng/mL. This result was also achieved by making some minor adjustments to the extraction protocol, such as overnight digestion of the tissue and genomic DNA elution with only 100 mL buffer.

The recovery of DNA from a biological source that minimizes stress and eliminates surgical risk for the bird supports the idea of Peniche et al. [23] that molecular sexing is the only efficacious technique for sexing certain avian species, such as golden eagle chicks, especially when overlapping biometric measures cannot be used to differentiate between sexes at an early age.

The source of DNA extraction is a key point in determining the eligibility of the molecular approach for sex determination. Additionally, the employment of avian sex-specific primers eliminates the possibility of contaminant DNA amplification deriving from naturally present microorganisms. Peniche et al. [23] also proposed such an approach, but the extraction of DNA from blood or oral sampling, compared to that from down or feather, is more invasive and stressful if applied to very young individuals [24].

Therefore, the results of this study could provide a guideline for species that have never been sexed before (18% of all species that we sexed), which also include species that, although dimorphic as adults (such as bird of prey), cannot be differentiated by sex in their early stage. This is also the case for *Accipiter nisus*, which shows clear dimorphism in adulthood, when males show a specific orange plumage on the chest and flanks [25], but during the first six to eight months of age, even the laparoscopic observation of the gonads cannot help because the ovaries may resemble testicles [26].

Until now, several molecular approaches have been proposed to sex the majority of avian species, but their costly and time-consuming procedure have resulted in low implementation.

Some species included in this study had already been sexed by other authors. Canon et al. [27] employed cytofluorimetry in *Amazona amazonica*, *Melopsittacus undulatus*, and *Nymphicus hollandicus* with limitations due to overlapping DNA weights between the two genders and the failure of intercalating dye as a result of degradation. Elnomrosy et al. [28] employed loop-mediated isothermal amplification to sex some species of the *Psittacidae*, *Cacatuidae*, and *Psittaculidae* families. The method appears time-consuming and is not widely applicable because it needs specific settings for almost every species. Horng et al. [29] used random amplified polymorphic DNA (RAPD-PCR) in *Columba livia*, and although it does not require a known prior sequence of template, it needs careful planning for each species. Hence, RAPD using short primers to produce DNA fragments by PCR is affected by low reproducibility and sensitivity to reaction conditions and can be a species-specific method, as evidenced by Griffiths and Tiwari [30]. Also, the nuclear microsatellite amplification using DNA from feathers can produce errors if the DNA is highly fragmented or is in low concentrations, as demonstrated by Mills et al. [31]. The amplified fragment length polymorphism (AFLP) method requires acrylamide gels, radioactive markers, or primers purified by HPLC, so it becomes an expansive technique [32].

By contrast, the molecular approach utilized in this study has proved effective in sexing all tested species. A further point confirming this is the fact that all pairs formed following sexing, housed in the same cage, and reaching sexual maturity during this study, produced offspring. These represented about 70% of all sexed species. The method could be applied to other species, starting with amplification by P2/P8 and continuing with RFLP if the simple PCR does not discriminate against gender. Obviously, mutations at the level of the consensus region of the employed primers or at the restriction sites for HaeIII and Asp 700I are possible limitations of this approach, but this is the limit of all molecular techniques.

## 5. Conclusions

This is the first report showing that a simple molecular approach can be used to successfully sex more than 150 avian species. We demonstrated the usefulness of down retrieved from chicks as a source of sufficient DNA obtained by the optimization of its extraction procedure. The use of the well-established P2/P8 primers for PCR, eventually combined with the RFLP, allowed for the correct sexing of all juvenile birds. This means that researchers involved in reintroduction projects and the preservation of endangered birds, as well as professional or amateur breeders of pet birds, will find this publication a guide for determining the sex of newborns to facilitate early pair formation. With this fairly simple and inexpensive technique, zoos and wildlife rescue centers can easily identify bird pairs for captive breeding programs. The invasiveness of down and feather plucking, although moderate, remains a specific limitation of this protocol. Nonetheless, it is necessary if one wants to eliminate errors in sex assignment and, thus, in pair formation. This sexing protocol, if tested in bird species belonging to the other orders, could be a candidate to become the first applicable to sex almost all birds.

## Figures and Tables

**Table 1:** List of the birds included in this study. After defining the size of the amplicons and/or restriction fragments in each adult, young individuals were sexed. The approximate length of the amplicon obtained by PCR or RFLP-PCR after electrophoresis analysis is shown in the appropriate column. The shaded row indicates the species molecularly sexed for the first time in this study.

ORDER	SPECIES	♂ P2/P8	♀ P2/P8	♂ HaeIII	♀ HaeIII	♂ Asp 700I	♀ Asp 700I
Accipitriformes	*Accipiter gentilis*	390	390	290 + 100	390 + 290 + 100		
	*Accipiter nisus*	390	390	330 + 60	390 + 330 + 60		
	*Aquila chrysaetos*	390	390	320 + 70	390 + 320 + 70		
	*Aquila rapax*	380	380	330 + 50	380 + 330 + 50		
	*Aquila spilogaster*	380	380	330 + 50	380 + 330 + 50		
	*Buteo buteo*	390	390	320 + 70	390 + 320 + 70		
	*Cathartes aura*	380	400 + 380				
	*Circaetus gallicus*	390	390	320 + 70	390 + 320 + 70		
	*Circus aeruginosus*	390	390	330 + 60	390 + 330 + 60		
	*Circus pygargus*	390	390	320 + 70	390 + 320 + 70		
	*Falco eleonorae*	390	390	330 + 60	390 + 330 + 60		
	*Gypohierax angolensis*	380	390 + 380				
	*Gyps africanus*	390	390	330 + 60	390 + 330 + 60		
	*Gyps fulvus*	390	390	330 + 60	390 + 330 + 60		
	*Gyps rueppelli*	390	390	330 + 60	390 + 330 + 60		
	*Haliaeetus albicilla*	380	380	340 + 40	380 + 340 + 40		
	*Hieraaetus pennatus*	380	380	330 + 50	380 + 330 + 50		
	*Milvus milvus*	390	390	320 + 70	390 + 320 + 70		
	*Neophron percnopterus*	370	380 + 370				
	*Parabuteo unicinctus*	390	390	330 + 60	390 + 330 + 60		
	*Pernis apivorus*	380	390 + 380				
	*Sarcogyps calvus*	400	400	320 + 80	400 + 320 + 80		
	*Sarcoramphus papa*	380	400 + 380				
Anseriformes	*Alopochen aegyptiaca*	360	370 + 360				
	*Anas platyrhynchos*	380	380	320 + 60	380 + 320 + 60		
	*Aythya fuligula*	360	370 + 360				
	*Chauna chavaria*	370	370	370	370	260 + 110	370 + 260 + 110
	*Cygnus atratus*	370	380 + 370				
	*Netta rufina*	360	380 + 360				
	*Oxyura leucocephala*	370	380 + 370				
	*Spatula querquedula*	370	380 + 370				
Apodiformes	*Apus apus*	380	380	330 + 50	380 + 330 + 50		
Bucerotiformes	*Aceros nipalensis*	380	380	320 + 60	380 + 320 + 60		
	*Bucorvus leadbeateri*	390	390	330 + 60	390 + 330 + 60		
	*Upupa epops*	380	390 + 380				
Charadriiformes	*Actitis hypoleucos*	370	380 + 370				
	*Chroicocephalus ridibundus*	360	380 + 360				
	*Burhinus oedicnemus*	360	380 + 360				
	*Ichthyaetus melanocephalus*	380	380	310 + 70	380 + 310 + 70		
	*Himantopus himantopus*	380	380	340 + 40	380 + 340 + 40		
	*Numenius arquata*	380	390 + 380				
	*Scolopax rusticola*	370	380 + 370				
	*Vanellus vanellus*	390	390	320 + 70	390 + 320 + 70		
Ciconiiformes	*Ciconia ciconia*	390	390	320 + 70	390 + 320 + 70		
Columbiformes	*Columba livia*	350	370 + 350				
	*Columba livia domestica*	350	370 + 350				
	*Streptopelia decaocto*	390	390	300 + 90	390 + 300 + 90		
Coraciiformes	*Alcedo atthis*	370	390 + 370				
	*Coracias garrulus*	380	390 + 380				
	*Dacelo leachii*	360	380 + 360				
	*Merops apiaster*	380	380	340 + 40	380 + 340 + 40		
Cuculiformes	*Cuculus canorus*	380	380	320 + 60	380 + 320 + 60		
Falconiformes	*Falco vespertinus*	390	390	330 + 60	390 + 330 + 60		
	*Caracara plancus*	380	400 + 380				
	*Falco ardosiaceus*	400	400	350 + 50	400 + 350 + 50		
	*Falco biarmicus*	380	380	330 + 50	380 + 330 + 50		
	*Falco cherrug*	380	390 + 380				
	*Falco jugger*	390	390	330 + 60	390 + 330 + 60		
	*Falco naumanni*	400	400	350 + 50	400 + 350 + 50		
	*Falco peregrinus*	380	390 + 380				
	*Falco subbuteo*	400	400	350 + 50	400 + 350 + 50		
Galliformes	*Alectoris barbara*	370	380 + 370				
	*Alectoris graeca*	370	380 + 370				
	*Coturnix coturnix*	390	390	330 + 60	390 + 330 + 60		
	*Pavo cristatus*	360	380 + 360				
	*Pavo muticus*	360	380 + 360				
	*Phasianus colchicus*	370	380 + 370				
	*Polypectron napoleonis*	380	380	310 + 70	380 + 310 + 70		
Gruiformes	*Balearica regulorum*	390	390	320 + 70	390 + 320 + 70		
	*Fulica atra*	380	380	340 + 40	380 + 340 + 40		
	*Grus grus*	390	390	320 + 70	390 + 320 + 70		
	*Rallus aquaticus*	380	390 + 380				
Otidiformes	*Otis tarda*	380	390 + 380				
Passeriformes	*Acridotheres tristis*	370	390 + 370				
	*Cardinalis cardinalis*	320	360 + 320				
	*Carduelis carduelis*	320	360 + 320				
	*Chloris chloris*	340	360 + 340				
	*Coloeus monedula*	370	390 + 370				
	*Corvus corone*	360	380 + 360				
	*Corvus corax*	360	380 + 360				
	*Erythrura psittacea*	370	390 + 370				
	*Gracula religiosa*	380	400 + 380				
	*Lanius collurio*	370	390 + 370				
	*Leiothrix lutea*	370	380 + 370				
	*Monticola solitarius*	380	380	330 + 50	380 + 330 + 50		
	*Oriolus oriolus*	370	370	290 + 80	370 + 290 + 80		
	*Parus major*	400	400	350 + 50	400 + 350 + 50		
	*Pica pica*	370	390 + 370				
	*Pinicola enucleator*	360	380 + 360				
	*Serinus serinus*	350	350	270 + 80	350 + 270 + 80		
	*Spinus atratus*	360	380 + 360				
	*Spinus magellanicus*	330	360 + 330				
	*Turdus iliacus*	360	380 + 360				
	*Turdus philomelos*	360	380 + 360				
	*Turdus pilaris*	370	390 + 370				
	*Turdus viscivorus*	360	380 + 360				
Pelecaniformes	*Ardea cinerea*	380	380	320 + 60	380 + 320 + 60		
	*Ardea purpurea*	380	380	320 + 60	380 + 320 + 60		
	*Egretta garzetta*	370	380 + 370				
	*Endocimus ruber*	390	390	340 + 50	390 + 340 + 50		
	*Geronticus eremita*	390	390	340 + 50	390 + 340 + 50		
	*Ixobrychus minutus*	390	390	330 + 60	390 + 330 + 60		
	*Nycticorax nycticorax*	390	390	330 + 60	390 + 330 + 60		
	*Pelecanus crispus*	400	400	330 + 70	400 + 330 + 70		
Phoenicopteriformes	*Phoenicopterus chilensis*	390	390	390	390	280 + 110	390 + 280 + 110
	*Phoenicopterus roseus*	390	390	390	390	280 + 110	390 + 280 + 110
Podicipediformes	*Podiceps cristatus*	370	380 + 370				
Psittaciformes	*Agapornis taranta*	360	380 + 360				
	*Agapornis fischeri*	360	380 + 360				
	*Agapornis personatus*	360	380 + 360				
	*Agapornis roseicollis*	360	380 + 360				
	*Alipiopsitta xanthops*	380	400 + 380				
	*Amazona amazonica*	390	400 + 390				
	*Amazona farinosa*	390	400 + 390				
	*Ara ararauna*	380	390 + 380				
	*Ara chloropterus*	380	390 + 380				
	*Aratinga solstitialis*	390	400 + 390				
	*Barnardius zonarius*	380	400 + 380				
	*Cacatua galerita*	380	380	320 + 60	380 + 320 + 60		
	*Cacatua sulphurea*	380	380	320 + 60	380 + 320 + 60		
	*Cyanoliseus patagonus*	370	390 + 370				
	*Eos bornea*	370	390 + 370				
	*Lorius garrulus*	370	390 + 370				
	*Melopsittacus undulatus*	380	400 + 380				
	*Myiopsitta monachus*	370	390 + 370				
	*Nandaius nenday*	370	390 + 370				
	*Nymphicus hollandicus*	360	380 + 360				
	*Pionites leucogaster*	370	390 + 370				
	*Pionus senilis*	370	390 + 370				
	*Platycercus elegans*	380	390 + 380				
	*Platycercus eximius*	380	390 + 380				
	*Poicephalus senegalus*	370	390 + 370				
	*Psittacula eupatria*	370	390 + 370				
	*Psittacula krameri*	370	390 + 370				
	*Psittacus erithacus*	370	390 + 370				
	*Pyrrhura frontalis*	370	390 + 370				
	*Pyrrhura picta*	370	390 + 370				
	*Trichoglossus haematodus*	370	390 + 370				
	*Trichoglossus moluccanus*	370	390 + 370				
Sphenisciformes	*Spheniscus humboldti*	370	380 + 370				
Strigiformes	*Asio flammeus*	390	390	320 + 70	390 + 320 + 70		
	*Asio otus*	390	390	320 + 70	390 + 320 + 70		
	*Athene noctua*	380	380	320 + 60	380 + 320 + 60		
	*Bubo africanus*	380	380	320 + 60	390 + 320 + 60		
	*Bubo bubo*	390	390	320 + 70	390 + 320 + 70		
	*Bubo virginianus*	380	380	320 + 60	390 + 320 + 60		
	*Otus scops*	380	380	320 + 60	380 + 320 + 60		
	*Strix aluco*	360	370 + 360				
	*Strix rufipes*	360	370 + 360				
	*Strix uralensis*	360	370 + 360				
	*Tyto alba*	390	390	320 + 70	390 + 320 + 70		
Suliformes	*Phalacrocorax carbo*	390	390	340 + 50	390 + 340 + 50		

## Data Availability

The data presented in this study are available on request from the corresponding author.
